# Production, Purification, and Characterization of Thermostable Alkaline Xylanase From *Anoxybacillus kamchatkensis* NASTPD13

**DOI:** 10.3389/fbioe.2018.00065

**Published:** 2018-05-15

**Authors:** Punam Yadav, Jyoti Maharjan, Suresh Korpole, Gandham S. Prasad, Girish Sahni, Tribikram Bhattarai, Lakshmaiah Sreerama

**Affiliations:** ^1^Molecular Biotechnology Unit, Nepal Academy of Science and Technology, Khumaltar, Nepal; ^2^Central Department of Biotechnlogy, Tribhuvan University, Kirtipur, Nepal; ^3^Microbial Type Culture Collection, Institute of Microbial Technology, Chandigarh, India; ^4^Department of Chemistry and Earth Sciences, Qatar University, Doha, Qatar

**Keywords:** thermostable, alkaline, anoxybacillus, xylanase, hot springintroduction

## Abstract

*Anoxybacillus kamchatkensis* NASTPD13 used herein as a source for thermostable alkaline xylanase were isolated from Paudwar Hot Springs, Nepal. NASTPD13 cultured at 60°C, pH 7 and in presence of inorganic (ammonium sulfate) or organic (yeast extract) nitrogen sources, produced maximum xylanase enzyme. Xylanase production in the cultures was monitored by following the ability of culture media to hydrolyze beech wood xylan producing xylooligosaccharide and xylose by thin layer chromatography (TLC). The extracellular xylanase was isolated from optimized *A. kamchatkensis* NASTPD13 cultures by ammonium sulfate (80%) precipitation; the enriched xylanase preparation was dialyzed and purified using Sephadex G100 column chromatography. The purified xylanaseshowed 11-fold enrichment with a specific activity of 33 U/mg and molecular weight were37 kDa based on SDS-PAGE and PAGE-Zymography. The optimum pH and temperature of purified xylanase was 9.0 and 65°C respectively retainingmore than 50% of its maximal activity over a broad range of pH (6–9) and temperature (30–65°C). With beech wood xylan, the enzyme showed Km 0.7 mg/ml and Vmax 66.64 μM/min/mg The xylanase described herein is a secretory enzyme produced in large quantities by NASTPD13 and is a novel thermostable, alkaline xylanase with potential biotechnological applications.

## Introduction

Lignocellulose, a major source of renewable organic matter, is mainly comprised ofof lignin, hemicellulose, and cellulose (Mmango-Kaseke et al., 2016). The lignocellulose is mainly obtained from agriculture, horticulture and forest waste, paper-pulp, timber and other agro-forest allied industries. Such lignocelluloses waste can potentially be utilized into various value-added products such as biofuels like bioethanol and biochemical products. (Pothiraj et al., 2006). The lignocellulosic bomass from nonedible feedstock provides many benefits such as (i) biomass being renewable and sustainable, (ii) carbon dioxide fixation in the atmosphere, (iii) facilitating local economic development, (iv) reducing air pollution from burning and rotting in fields, (v) providing energy security for countries dependent on imported oil, and (vi) creating high technology jobs. (Balan, 2014). Additionally, there are few advantages of using thermostable and alkali stable enzymes in industrial processes, primarily it increases the reaction rate, gives enzymes longer half life, decreases the possibility of microbial contamination as compared to mesophiles, lowers the chance of phage infection, improves the solubility of lignocellulosic substrates, recovery of volatile products, and increases the catalytic efficiency throughout industrial processes (Zamost et al., 1991; Ellis and Magnuson, 2012; Zeldes et al., 2015).

**Figure 1:**
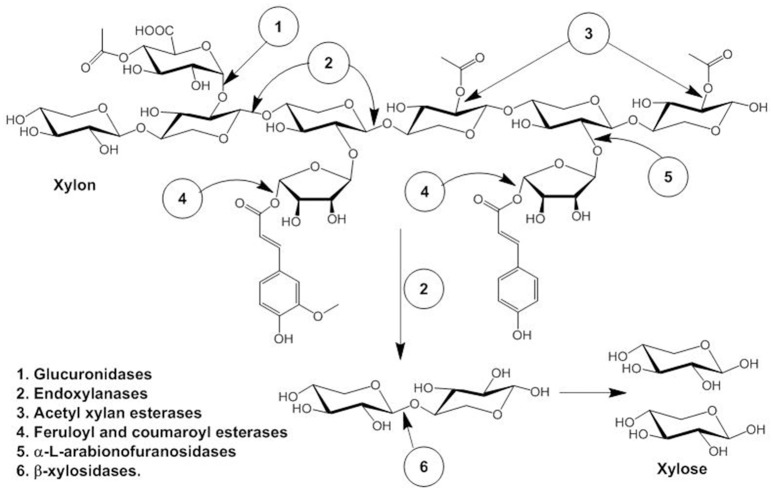
Hemicellulose degrading enzymes and their specificity of action.

Hemicellulose, the second most abundant polysaccharide after cellulose consists of β-1, 4 linked D-xylopyranosyl units linked with branches of O-acetyl, α-L-arabinofuranosyl and α-D-glucuronyl residues (La Grange et al., 2001). Synergistic action of several enzymes are required for complete degradation of hemicellulose to pentose sugar, namely endoxylanases (endo-β- 1,4-xylanase), β-xylosidases (xylan 1,4-β-xylosidase), and α- glucuronidases (α-glucosiduronase) and side-chain cleaving enzymes: α-L-arabinofuranosidase, feruloyl esterase, and acetyl xylan esterase that produces xylooligomers which are further degraded to monomeric sugar xylose by β-D-xylosidases (Ellis and Magnuson, 2012; Sun et al., 2012; Motta et al., 2013). The schematic diagram is explained in Figure 1. In short, xylans consist of D-xylose homopolymer linked through β-1,4 glycosidic linkage and xylanase (E.C 3.2.1.8) degrades β-1,4 glycosidic bond randomly producing xylose and xylo-oligosaccharides like xylobiose (Kamble and Jadhav, 2012). Therefore, xylanase plays a critical role in the degradation of hemicellulose, accordingly, has many industrial applications, e.g., bio-conversion of lignocellulosic materials to fermentable substrates in biofuel industries (Podkaminer et al., 2012; Roy et al., 2013).

The conversion of lignocellulosic feedstock has been major challenge in the process of biofuel production due to the recalcitrant nature of plant cell walls, enzyme efficiency, and biomass quality which lead researchers to continued discovery of novel thermostable enzymes in order to establish a better database of enzymes and identification of more efficient enzymes (Balan, 2014).

Thermophilic microorganisms are attractive candidates for biomass conversion and overcome a way to break of feedstock process-operating challenges (Mi et al., 2014).

Genus *Anoxybacillus* has been shown to secrete the variety of heat-stable lignocellulolytic enzymes such as cellulase and xylanase important in biomass degradation (Ellis and Magnuson, 2012). Some of the xylanases and xylosidases have already been characterized from *Anoxybacillus* (Goh et al., 2013), while there are still many predicted xylanolytic enzymes encoded by *Anoxybacillus* that are yet to be isolated and characterized. *Anoxybacillus kamchatkensis, first* reported from by Kevbrin et al. (2005) was isolated from the Geyser valley located in the Kamchatka peninsula (Far East region of Russia). However, to our knowledge further enzymatic characterization of *A. kamchatkensis* has not been reported so far. In this study, microorganisms producing xylanase were isolated from Paudwar hot spring located in Myagdi district, Nepal (Yadav et al., 2017b), and, from among them, one bacterial strain *A. kamchatkensis* NASTPD13 showing distinct xylanase activity (Yadav et al., 2017a) in the cell-free extract, was selected for purification and further characterization of the enzyme.

## Materials and methods

### Phenotypic characteristics *Anoxybacillus kamchatkensis* NASTPD13

Cell morphology of bacteria was studied by Gram's staining using the commercially available kit (HIMEDIA, Bangalore, Karnataka, India) and endospore staining as described by Mormak and Casida (1985). The detailed description of the bacterial species characterization has been described in a separate study (Yadav et al., 2017a).

### Culture conditions and xylanase production

The secretory xylanase enzyme was isolated from cultures of *A. kamchatkensis* NAST-PD13, a bacteria isolated from Paudwar hot spring of Myagdi, Nepal. Bacteria was cultured in 50 ml minimal salt medium (MSM) pH 7 containing 0.5 g/l NaNO_3_; 1.0 g/l K_2_HPO_4_; 0.5 g/l MgSO_4_.7H_2_O, 0.01g/l FeSO_4_.7H_2_O, and 1.0 g/l yeast extract supplemented with 0.5% Beech wood xylan (Sigma- Aldrich, St Louis, MO, USA) and incubated overnight in shaking incubatorat 60°C and 200 rpm (Padilha et al., 2015). At specified intervals, small aliquots of the cultures were withdrawn, centrifuged (12,000 × g for 10 min at 4°C), and supernatant was tested for xylanase activity.

The optimization of medium and culture conditions for maximum xylanase production was carried out by stepwise variation of physicochemical parameters of growth conditions of *A. kamchatkensis* NAST-PD13. Initially, the bacterial isolate was grown in various MSM containing 0.5% xylan as reported previously (Nakamura et al., 1993; Kacagan et al., 2008; Maki et al., 2011; Padilha et al., 2015) (data not shown). Nakamura and Horikoshi basal medium had been routinely used for the isolation of xylanase producing microorganisms. Further xylanase production and optimization were studied using the basal medium proposed by Nakamura et al. (1993) by varying ‘one factor at a time and keeping the rest factors constant. The factors and their effects on xylanase production assessed were temperatures (40–75°C), pH (4 to 11), concentration of beech wood xylan (0.0–3.0%) in MSM and nitrogen sources[KNO_3_, (NH_4_)_2_SO_4_, peptone, yeast extract and urea] as described previously (Irfan et al., 2016). All the experiments were performed in triplicate, and the data reported herein are the average of triplicate experiments.

### Xylanase enzyme assay

Xylanase activity was measured using xylan 1% (wt/v) in 100 mM sodium phosphate buffer, pH 7.0 as substrate. Xylose released from xylan was measured by 3′ 5′dinitrosalicylic acid (DNSA) method using xylose as a standard. One unit of endo-1, 4-β-xylanase was defined as “the amount of enzyme required to release 1 μmol of xylose per minute under standard assay conditions” previously described (Bhalla et al., 2015).

### Growth curve and xylanase activity

2.5 ml of overnight culture of *A. kamchatkensis* NASTPD13 was inoculated in 250 ml of MSM supplemented with 1% beech wood xylan and incubated in an orbital shaker at 60°C and 200 rpm. Aliquots of the culture were withdrawn at regular intervals for up to 48 h to measure the growth and secretion of xylanase. Bacterial growth was monitored by measuring absorbance at 600 nm (turbidity measurements). The aliquots after absorbance measurements were centrifuged (12,000 × g, 10 min) to remove all solids including the bacterial cells and supernatants were analyzed for xylanase activity as described above (Shang et al., 2013).

### Identification of hydrolysis products

Xylanase enzyme preparations (including MSM culture broth supernatants) were added in 50 ml of beech wood xylan suspension (1% of beech wood xylan in 50 mM sodium phosphate buffer pH 7.0), and incubated at 60°C with mild agitation (30 rpm) for 24 h. The hydrolyzed products were separated by thin layer chromatography (TLC; 0.25 mm layers of silica gel F 254 plates; Merck, India) and xylose release was detected using D-xylose as the reference standard. A mixture of chloroform/acetic acid/water (6:7:1 by volume) was used as the mobile phase. Sugar spots were detected by spraying the TLC plates with 5% H_2_SO_4_ in ethanol (95%) followed by drying the plates in a hot-air oven at 105°C for 10 min (Haddar et al., 2012).

### Estimation of total protein

Concentration of total protein was determined using a Pierce BCA protein assay kit (Thermo Scientific, Waltham, MA, USA) as described by manufacturers protocol using BSA protein as standard (Watanabe et al., 2014).

### Purification of xylanase

#### Ammonium sulfate precipitation

The xylanase enzyme content in the culture broth was enriched by protein precipitation using (NH_4_)_2_SO_4_. NH_4_)_2_SO_4_was added to the culture broth via constant gentle stirring and the mixtures were then left overnight at 4°C to precipitate the protein, the protein precipitates were separated by centrifugation at 12,000 × g for 10 min at 4°C (Wingfield, 2001). The precipitate obtained was dissolved in 50 mM sodium phosphate buffer, pH 7.0 and dialyzed against the same buffer using Snakeskin pleated dialysis tubing (Molecular weight cut-off 3 kDa) for 24 h at 4°C. The dialyzed product was concentrated using Amicon® Ultra centrifugal filter units with molecular weight cut-off of 5 kDa (Millipore Corp, Bedford, USA) (Viet et al., 1991; Ninawe et al., 2008).

#### Column chromatography

The dialyzed and concentrated protein preparation (2 ml) was purified by gel filtration chromatography. Manually packing of Sephadex G100 column using 50 mM NaCl with a flow rate of 1.0 ml/min was done. The fractions showing xylanase activity were collected and concentrated. Subsequently, salt was removed by dialysis using a Float-A-Lyzer G2 system (molecular mass cut-off, 0.5 to 1 kDa; Spectrum Laboratories, USA) (Baindara et al., 2015).

### SDS-PAGE and zymography

SDS-PAGE was performed as described by Laemmli (1970). Gels were stained for proteins with Coomassie brilliant blue R-250. Excess stain from the gels was removed by repeatedly soaking the gels with a de-staining solution (1:1:8 mixture of methanol: glacial acetic acid: distilled water by volume). The molecular mass of xylanase was determined by comparing the electrophoretic mobility of purified xylanase to relative mobility of the reference protein marker (PageRuler, Prestained Protein Ladder, ThermoScientific, USA). The gels were stained for enzyme activity (zymogram analysis) by in-gel Zymography technique. Briefly, SDS-PAGE gels were copolymerized with the substrate (0.2% beech wood xylan). After electrophoresis, the gels were leached to remove SDS by soaking them with 2.5% Triton X-100 non-ionic detergent for 1 h to allow for partial refolding of enzymes to their active conformation. Subsequently, the gel was incubated in 100 mM phosphate buffer (pH 6.8), at 55°C for 1 hr, to allow the enzymes to digest the copolymerized substrate (Vandooren et al., 2013). Gels were then stained with 0.1% Congo red for 10 min and destained with 1 M NaCl until zones of clearing were seen. The gels were fixed with 5% acetic acid and photographed (Kacagan et al., 2008).

### Characterization of NASTPD13 xylanase

All of the physicochemical characterization studies reported herein was performed using purified xylanase enzyme preparation.

### Effect of temperature on activity and stability

The effect of temperature on enzyme activity was determined by DNSA assay as described above in the temperature ranging from 30 to 80°C, Aliquots of purifiedenzyme were incubated for 24 h at various temperatures from 30 to 80°C with 5°C increments. Post incubation, the tubes were removed and rapidly cooled in an ice bath and residual enzyme activity were determined. The percentage of relative xylanase activity was calculated by comparing them to the enzyme activities in enzyme preparations that were not heat-treated (Gaur and Tiwari, 2015).

### Effect of pH on activity and stability

The purified enzyme was incubated with 1% beech wood xylan substrate prepared in buffers solutions with pH ranging from 3 to 11 at 60°C for 30 min (Raj et al., 2013). The buffers used for this purpose were sodium citrate buffer (50 mM, pH 3–6), sodium phosphate buffer (50 mM, pH 6–8), Tris-HCl (50 mM, pH 8–9) and glycine–NaOH buffer (50 mM, pH 9–11). Stability of the enzyme at different pH values was studied by incubating 100 μl of the purified enzyme at various pH ranging from 4.0–11.0 for 24 h at 65°C and then residual xylanase activity was determined by DNSA assay (Gaur and Tiwari, 2015).

### Enzyme kinetics

The effects of substrate concentration ranging from 0.05 to 5% (w/v) beech wood xylan as the substrate were evaluated under standard condition. Km amd Vmax were obtained from Lineweaver-Burk method (Genc et al., 2015).

### Shelf life of enzyme

The purified enzymes were kept in refrigerator (4°C) and room temperature (25°C). Samples were withdrawn each day up to 6 weeks and the residual xylanase activities were determined (Sharma and Chand, 2012).

## Results

Thermostable xylanase optimally active at high temperature as well as broad pH range has gain the industrial importance as it can handle the harsh processing conditions (Turner et al., 2007; Bhalla et al., 2015). Describe herein is one such xylanase we have isolated from *A. kamchatkensis* NASTPD13 and compared its properties to other xylanases previously reported. Given the results described herein, we are confident that this is the first study of its kind that reports the isolation and characterization of novel xylanase from *A. kamchatkensis* NASTPD13.

### Description of *A. kamchatkensis* NASTPD13

**Figure 2:**
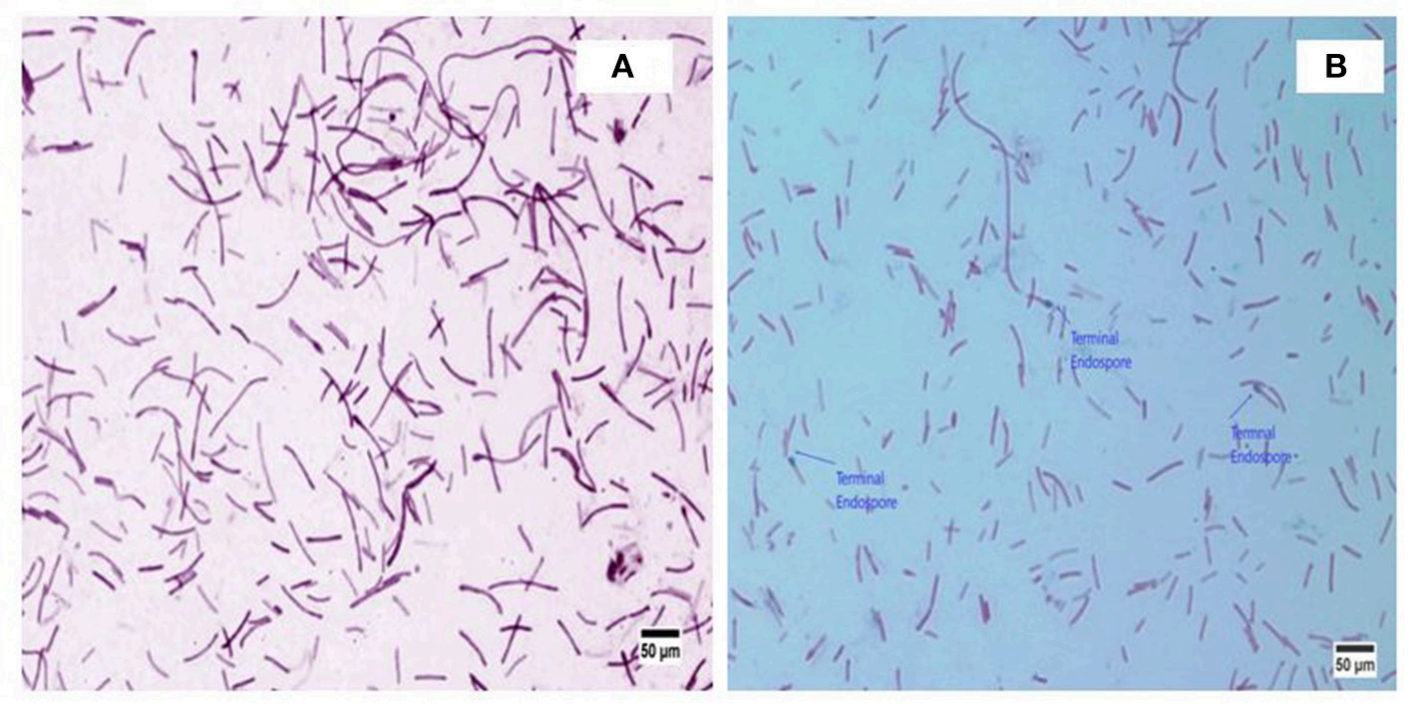
Morphological image of *A. kamchatkensis* NASTPD13 **(A)** Gram staining of *Anoxybacillus kamchatkensis* NASTPD13 showing blue color rod shape bacilli. **(B)** Endospore staining of *Anoxybacillus kamchatkensis* NASTPD13 showing terminal oval shape endospore.

**Figure 3:**
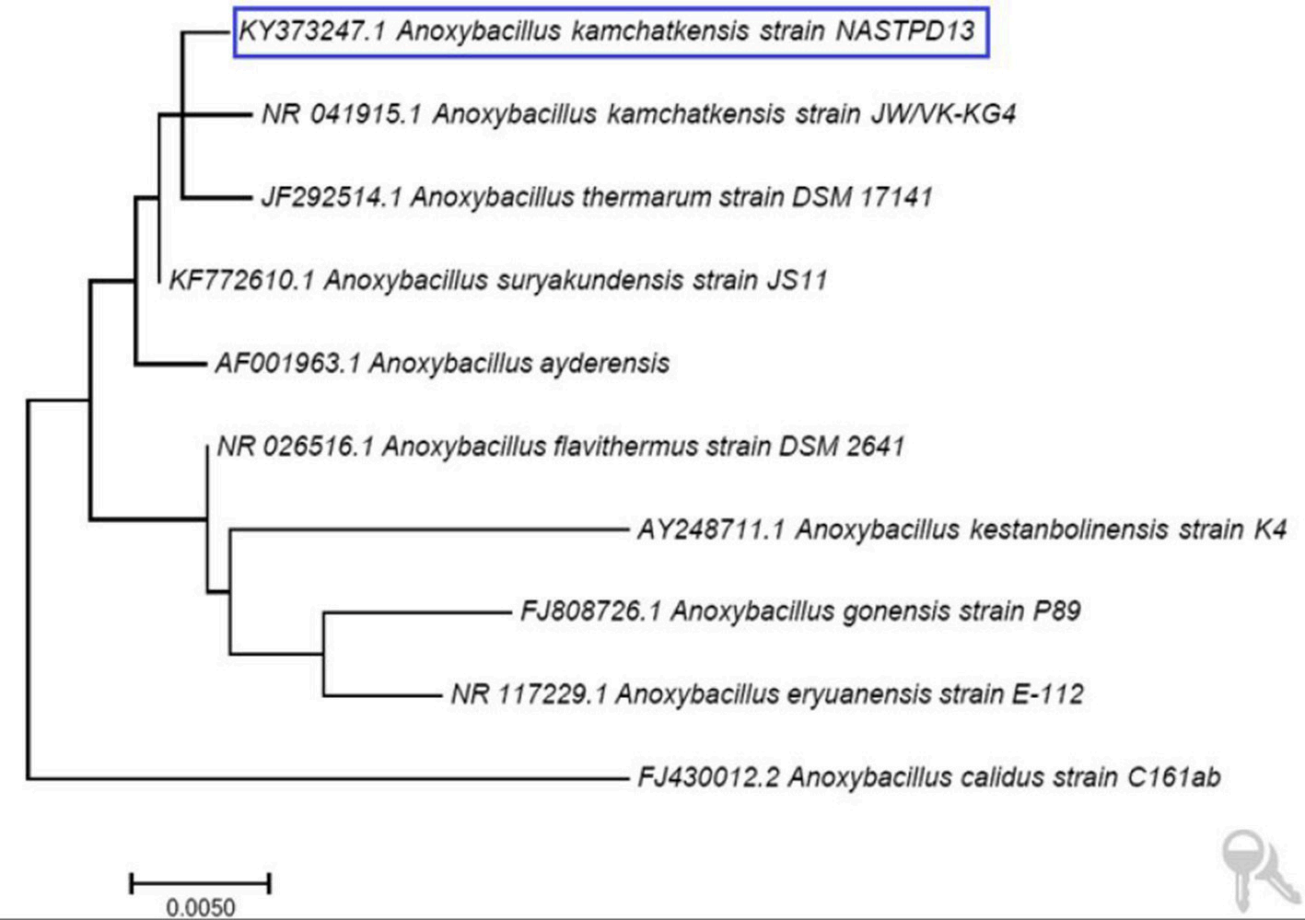
Phylogenetic tree based on 16S rDNA sequence of *A. kamchatkensis* NASTPD13 The evolutionary history was inferred by using the Maximum Likelihood method based on the Kimura 2-parameter model by using MEGA7.

*A. kamchatkensis* NASTPD13 colonies were 1-2 mm in diameter, cream colored, and regular in shape with round edges (Yadav et al., 2017a). The strain was found to be a straight or slightly curved, rod-shaped, facultative, Gram-positive bacteria (Figure 2A) with terminal spore forming (Figure 2B). It grew over a wide range of pH (5.0–11) and temperature (37–75°C). The other strains of *A. kamchatkensis*, e.g., *A. kamchatkensis* JK/VK-W4 utilize glucose, fructose, and trehalose, in aerobic conditions butshows no catalase/oxidase activity, and no growth on starch and raffinose (Kevbrin et al., 2005). In contrast *A. kamchatkensis* NASTPD13 (this study) was catalase and oxidase positive, able to grow on starch and raffinose, glucose and fructose in aerobic conditions. 16S rDNA sequence alignment of *A. kamchatkensis* NASTPD13 and BLAST analysis showed highest similarity with *A. kamchatkensis* JK7/VK-KG4 (Figure 3; Kevbrin et al., 2005). Therefore, based on biochemical, morphological characteristics and 16S rDNA sequences *A. kamchatkensis* NASTPD13 was identified as *A. kamchatkensis* and designated as *A. kamchatkensis* NASTPD13 and the16S rDNA sequence was submitted to Gene Bank (Accession No. KY373247).

### Optimization of culture conditions for xylanase production

**Figure 4:**
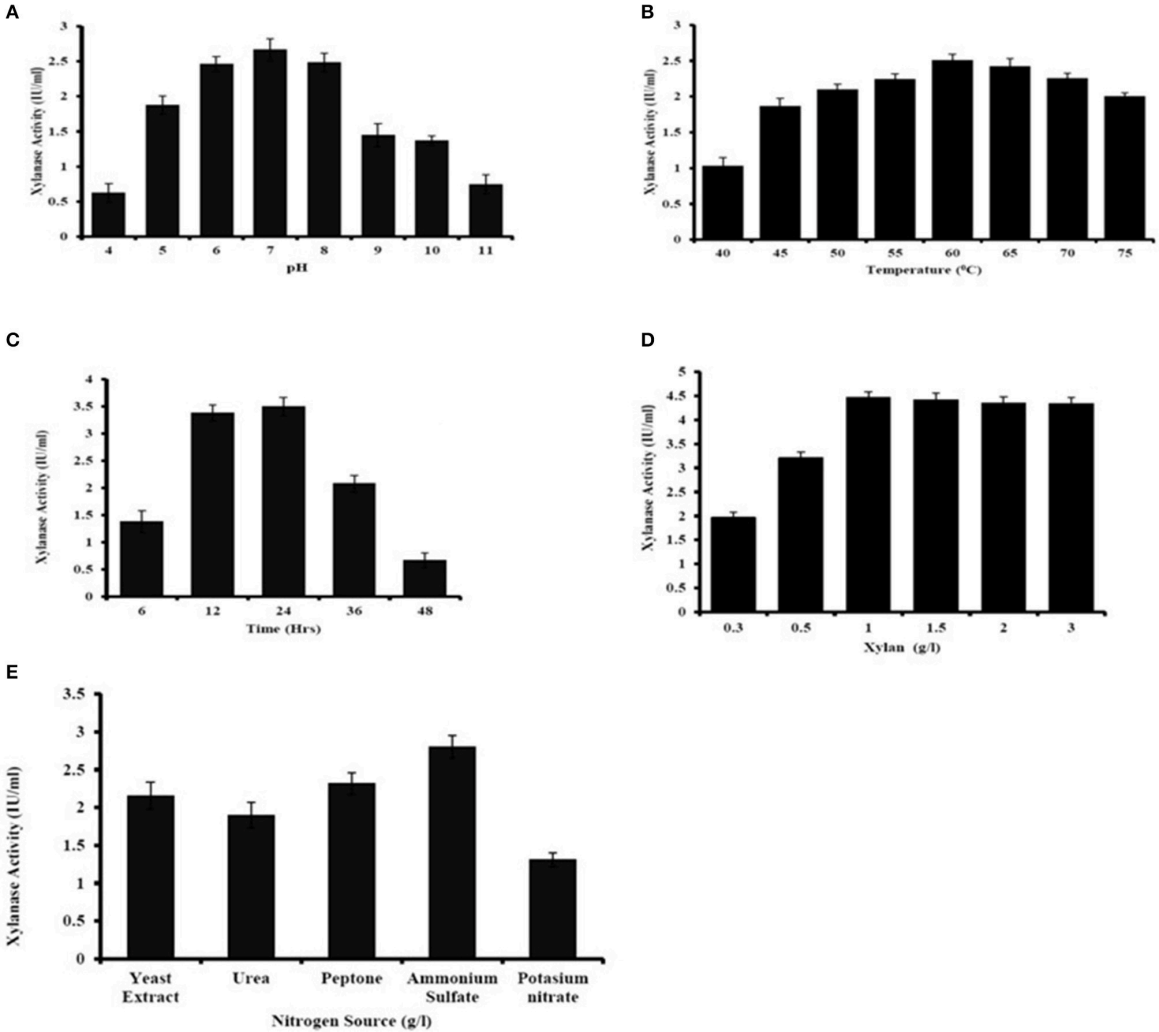
Optimization of culture condition for xylanase production from *Anoxybacillus kamchatkensis* NASTPD13 **(A)** pH **(B)** Temperature **(C)** Incubation time **(D)** Xylan concentration **(E)** Nitrogen sources. Xylanase production measured by its activity as described in Materials and Methods. The bacteria were grown in MSM medium at pH 7 and 60°C for 48 h unless the culture condition was differed as indicate in the Figure. Results are the mean of triplicate experiments with ± standard deviation represented by error bars.

The major parameters, *viz*, incubation time, pH, temperature, substrate concentration, nitrogen source in medium for xylanase production was optimized (Figure 4). The maximum xylanase activity was seen when the bacteria were grown at pH 7 and decreased in enzymatic activity were seen below or above the optimum pH (Figure 4A). The optimum temperature for xylanase production was found to be at 60°C and there was decrease in xylanase production below 55°C and increased above 65°C (Figure 4B). Age of culture also affected the production of xylanase activity. The result showed that after 24 h of incubation there was the decrease in xylanase production by *A. kamchatkensis* NASTPD13 (Figure 4C). Substrate concentration is crucial part for maximum xylanase production. Maximum xylanase production by *A. kamchatkensis* NASTPD13 was obtained at 1% xylan concentration (Figure 4D) further increase in substrate concentration reduced the enzyme production. Among all the tested inorganic and organic nitrogen sources, ammonium sulfate, yeast extract, and peptone were found best for NAST-PD13 growth and xylanase production (Figure 4E).

### Growth curve and xyalanase activity

**Figure 5:**
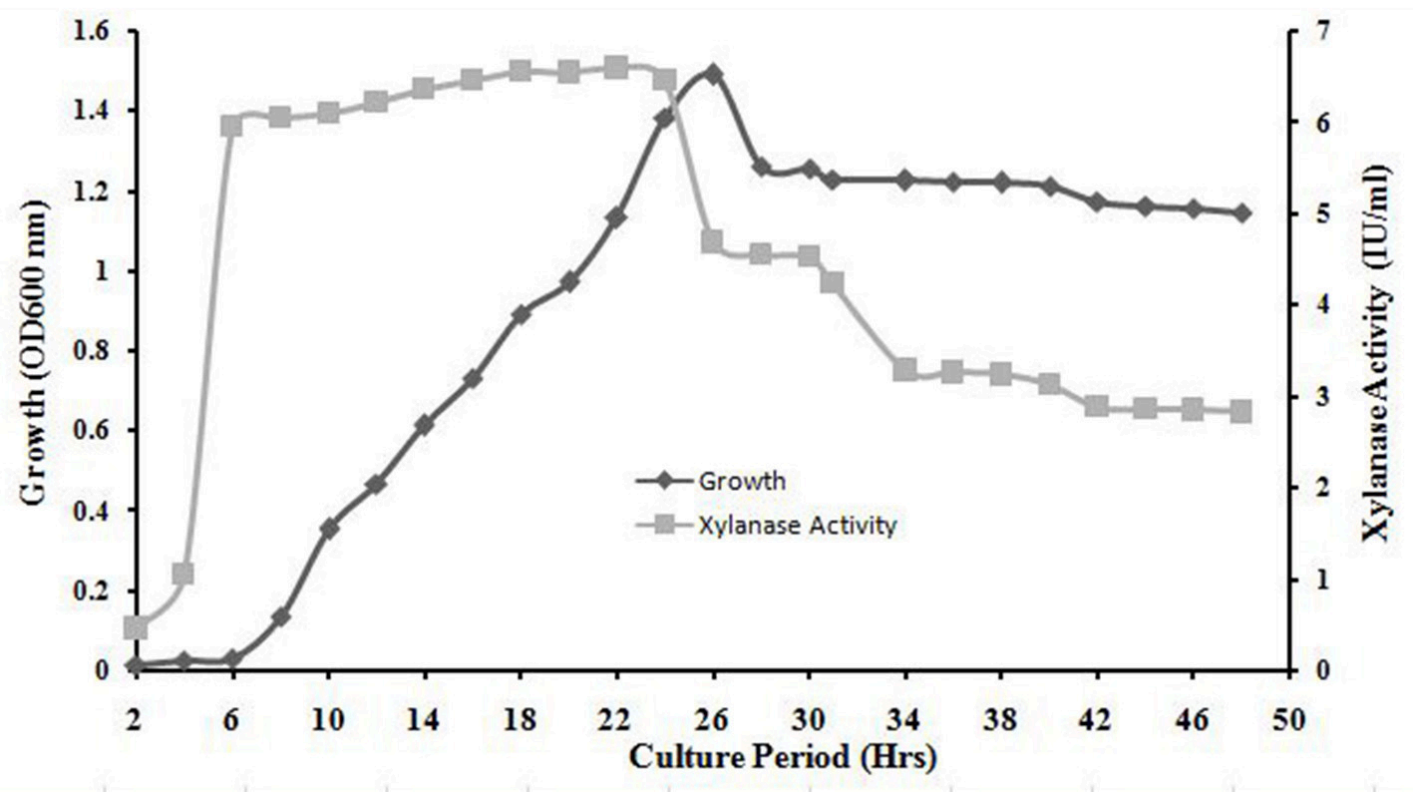
Bacterial growth curve and time course of *A. kamchatkensis* NASTPD13 xylanase production. The bacteria were cultured under optimal growth conditions described in Materials and Methods and the broth was tested for secretory xylanase at intervals of 2 h for up to 50 h.

The exponential growth of *A. kamchatkensis* NASTPD13 started after 6 h of incubation and ended at 26 h. The stationary phase began at 26 h. The absorbance at the stationary phase is ~1.26 and maximum values reached ~1.49 in 26 h (Figure 5). Xylanase production increased rapidly in an early growth phase of 0 to 6 h, remained more or less constant from 6 to 24 h, with a maximum value of 6.56 U/ml at 18 h. After 24 h there was a decrease in xylanase activity (Figure 5).

### Analysis of hydrolytic product

**Figure 6:**
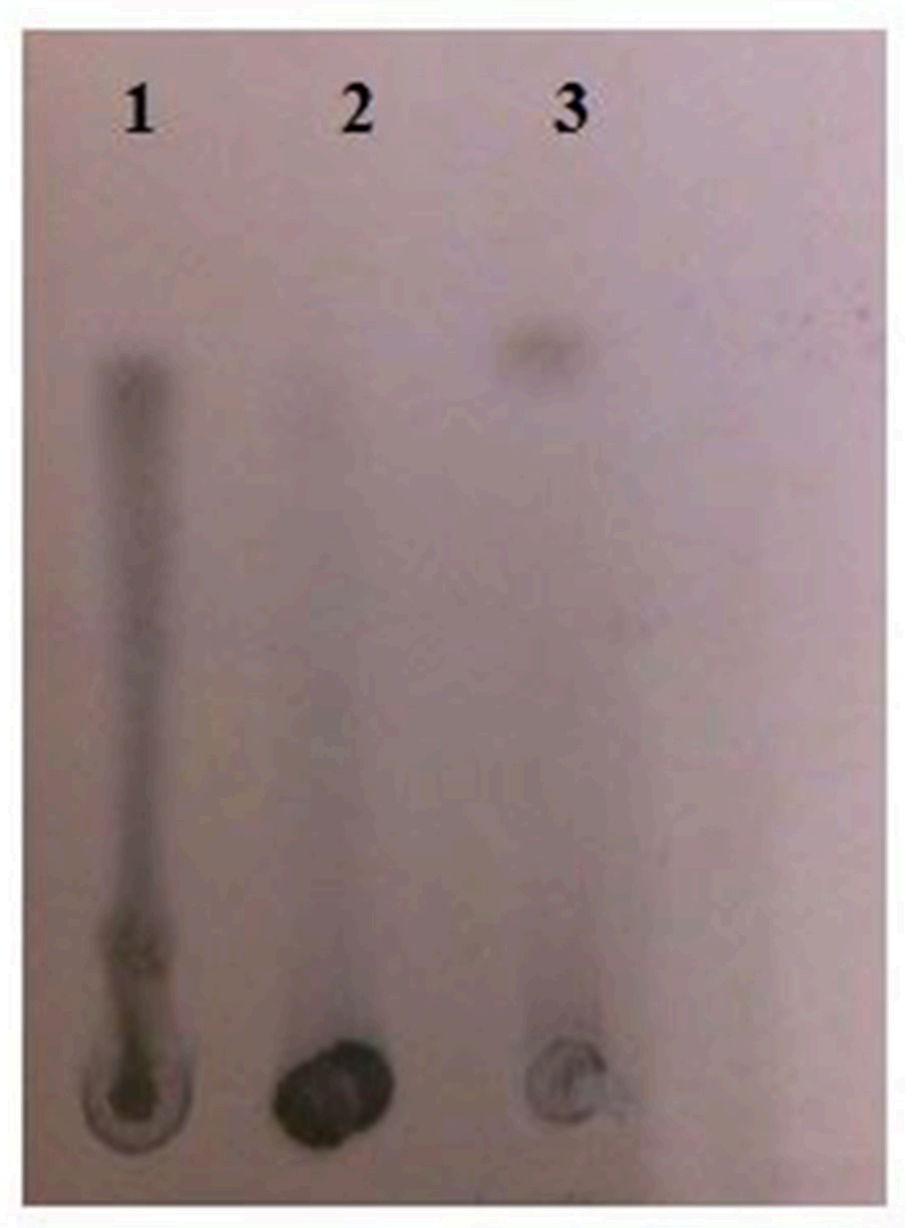
Thin layer chromatography (TLC) of xylan hydrolyzed by *A. kamchatkensis* NASTPD13 xylanase (Lane 1); Unhydrolyzed Xylan (Lane 2); D-Xylose standard (Lane 3).

Before the purification of enzyme, hydrolytic activity of crude xylanase was examined using beech wood xylan. TLC of enzymatic hydrolysis products (Figure 6) showed that crude xylanase from *A. kamchatkensis* NASTPD13 cleaved beech wood xylan backbone to liberate xylooligosaccharide and small amounts of xylose.

### Purification of xylanase

#### Ammonium sulfate and column chromatography

The culture filtrate precipitated by 80% ammonium sulfate had maximum xylanase activity. The ammonium sulfate enriched fraction of xylanase was dialyzed and the dialyzed fraction was further subjected to Sephadex G100 gel-permeation column chromatography. The fractions showing xylanase activity were pooled, concentrated, and analyzed by SDS-PAGE. The protein concentration and xylanase activity determined at each purification step showed an increase in specific activity from 6.32 to 16.49 U/ml and resulted in 11-fold purification (Table 1). SDS-PAGE analysis of the final faction showed a single band. The procedure used herein for purifications is simple, efficient and cost-effective for enzyme production for industrial processes.

**Table 1:** Purification of xylanase from *A. kamchatkensis* NASTPD13 Summary^*^.

**Purification step**	**Xylanase (U/ml)**	**Protein (mg/ml)**	**Specific Activity (U/mg)**	**Purification fold**
Crude	6.32	2.1	3.01	1
80% Ammonium sulfate pool	7.00	1.8	3.89	1.3
Dialyzed pool	7.15	1.2	5.96	2.0
Concentrated pool after dialysis	8.43	0.8	10.54	3.5
Sephadex G-100 pool	16.49	0.5	32.98	11.0

* *Purification was done by 80% Ammonium sulfate precipitation and the dialyzed and concentrated product was further passed through Sephadex G-100 column*.

### SDS page and zymography

**Figure 7:**
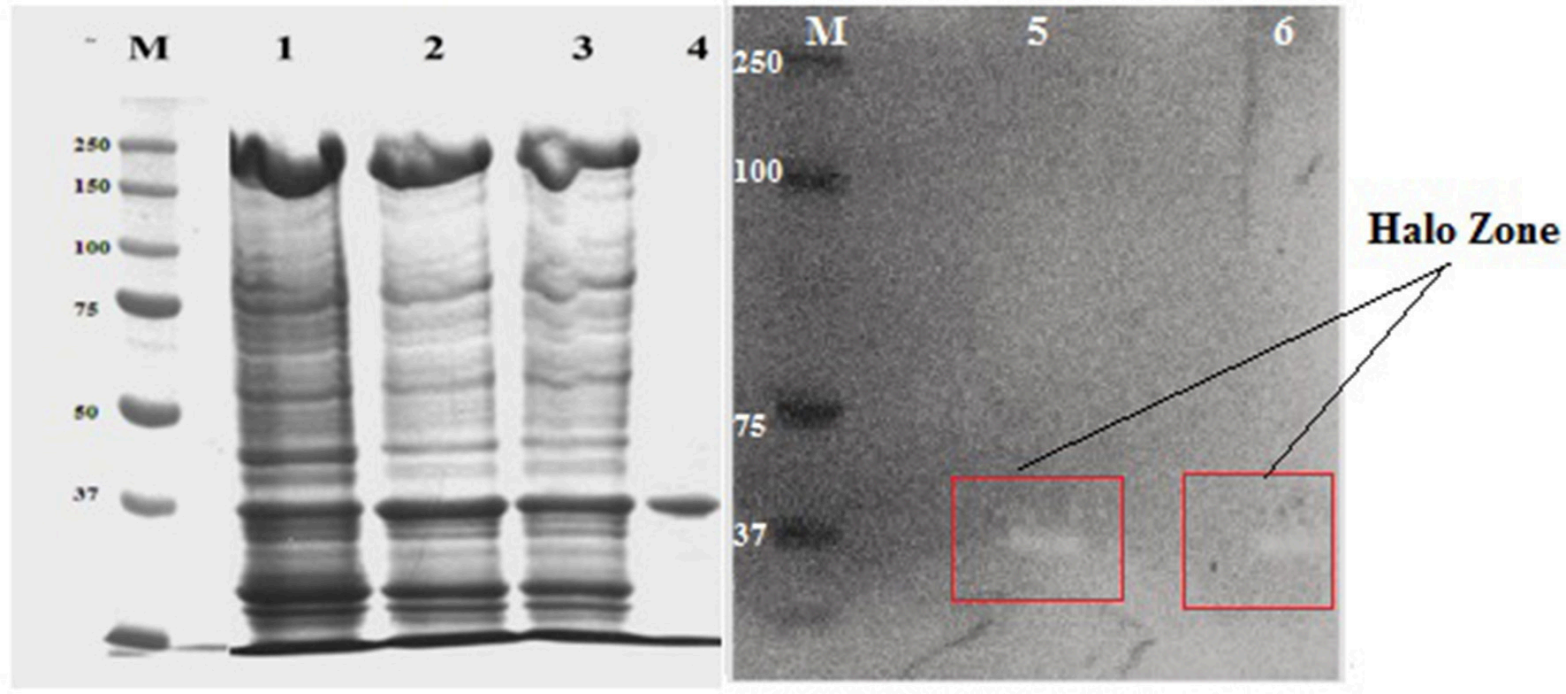
SDS-PAGE and zymogram analysis of purified NASTPD13 xylanase Purified fraction obtained during purification process were analyzed by SDS-PAGE; M: Protein molecular weight markers (PageRuler, Prestained Protein Ladder, ThermoScientific, USA), 1, Crude enzyme; 2, Ammonium sulfate precipitate; 3, Amicon filter concentrated sample; 4, Sephadex G-100 product; 5 and 6, Zymogram; Xylanase activity of NASTPD13.

The molecular mass of purified xylanase, as judged by SDS-PAGE, was found to be 37 kDa (Figure 7). Zymogram containing 0.2% beech wood xylan were performed to study the functional nature of the purified enzyme and the technique showed a prominent activity band corresponding to 37 kDa, showing the same electrophoretic motility which added a new element to supports the xylanase activity of the purified enzyme (Figure 6).

### Enzyme characterization

#### Effect of pH on enzyme activity and stability

**Figure 8:**
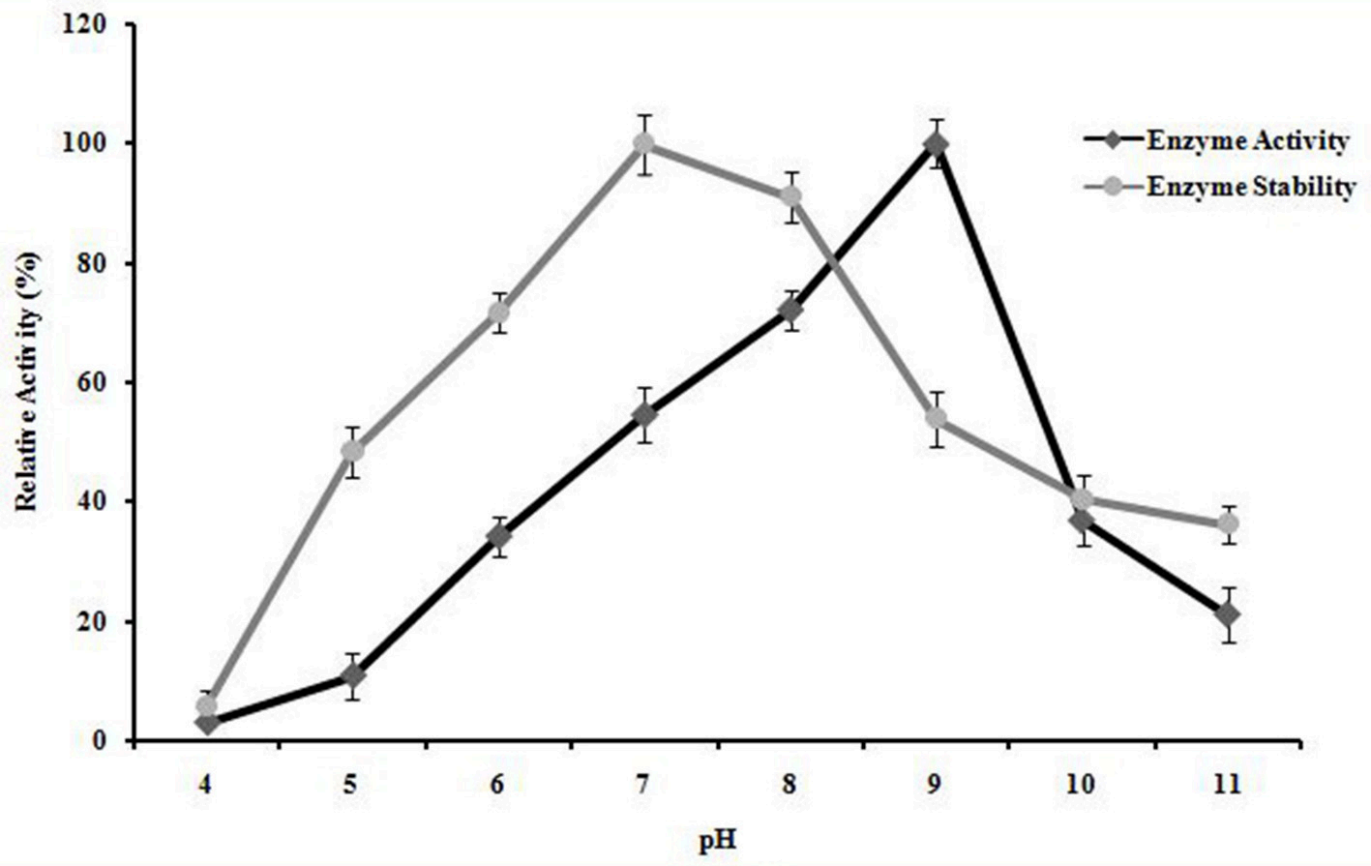
Effect of pH on enzyme activity and stability of *A. kamchatkensis* NASTPD13 xylanase. The values are means of three replicates at each temperature. The maximum activity measured under optical condition was defined as having the relative activity of 100%. The values are means of three replicates at each temperature. Results are the mean of triplicates with ± standard deviation represented by error bars.

*A. kamchatkensis* NASTPD13 xylanase showed enzyme activity over a broad range of pH (5.0–11) (Figure 8). The enzyme activity was found to be similar between pH 7 and 9, with maximum activity at pH 9. *A. kamchatkensis* NASTPD13 xylanase was more stable in the range of pH 6 to 9 and retained 71–100% of its maximal activity. On the other hand, above pH 9–11, the activity decreased from 53 to 36% of its original activity. *A. kamchatkensis* NASTPD13 xylanase retained 53.95% activity.

#### Effect of temperature on enzyme activity and stability

**Figure 9:**
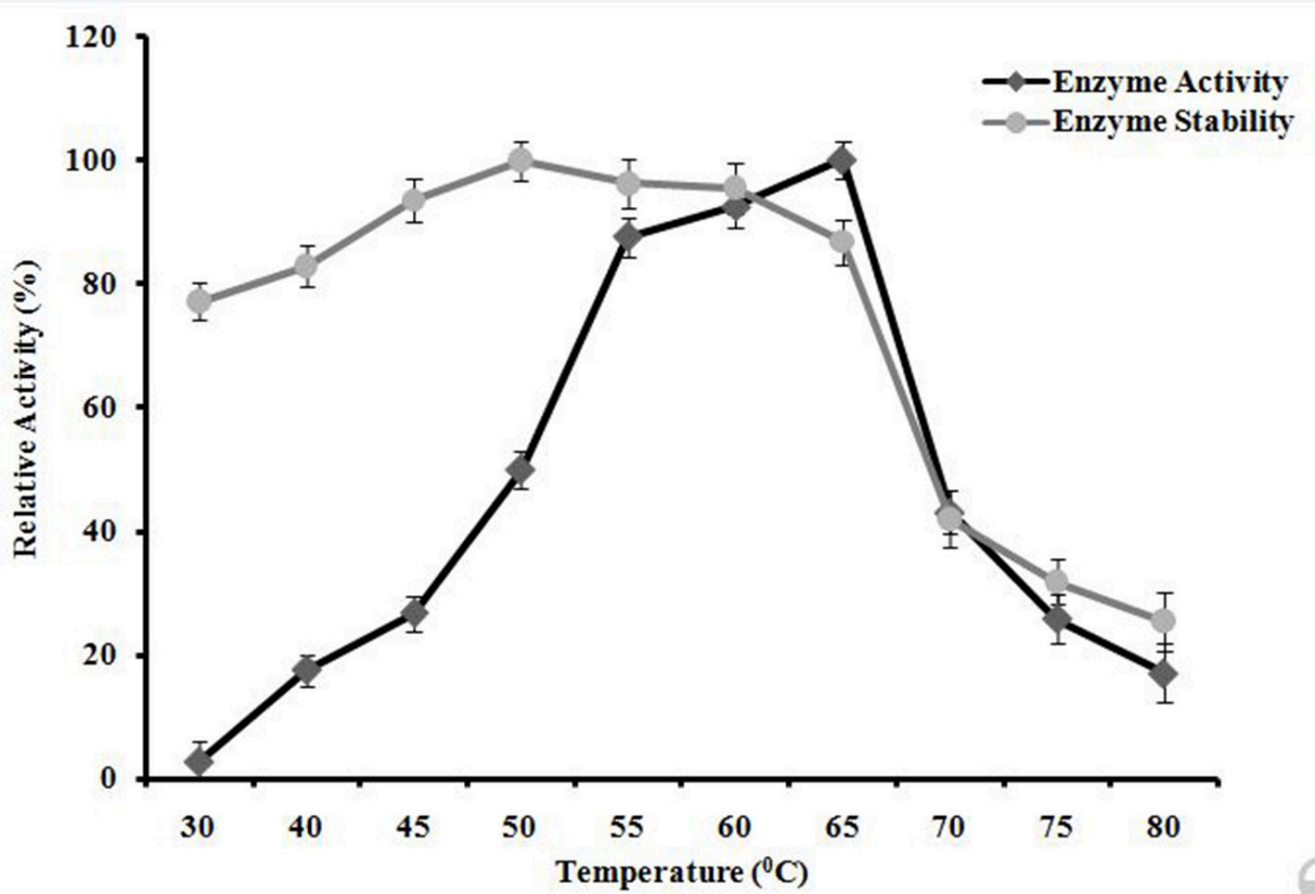
Effects of temperature on A. kamchatkensis NASTPD13xylanase enzyme activity. Measurement of stability of the enzyme incubated for 1 h at different temperatures. Maximum activity measured under optimal condition was defined as having relative activity of 100%. Values are the mean of triplicates with ± standard deviation represented by error bars.

*A. kamchatkensis* NASTPD13 xylanase activity increased with incubation temperature within the range of 30 to 65°C showing maximum activity when incubated at 65°C (Figure 9). At 70°C, a relative activity was 43% of maximum was seen. This decreased to a residual activity of about 17% at 80°C. *A. kamchatkensis* NASTPD13 xylanase was found thermostable at temperatures from 55 to 65°C (Figure 9), The enzyme showed 100, 96.30, 95.57, 86.85, 42.03, 31.85, 25.51% of residual activity after incubation at 50, 55,60, 65, 70, 75, and 80°C, respectively.

#### Enzyme kinetics

**Figure 10:**
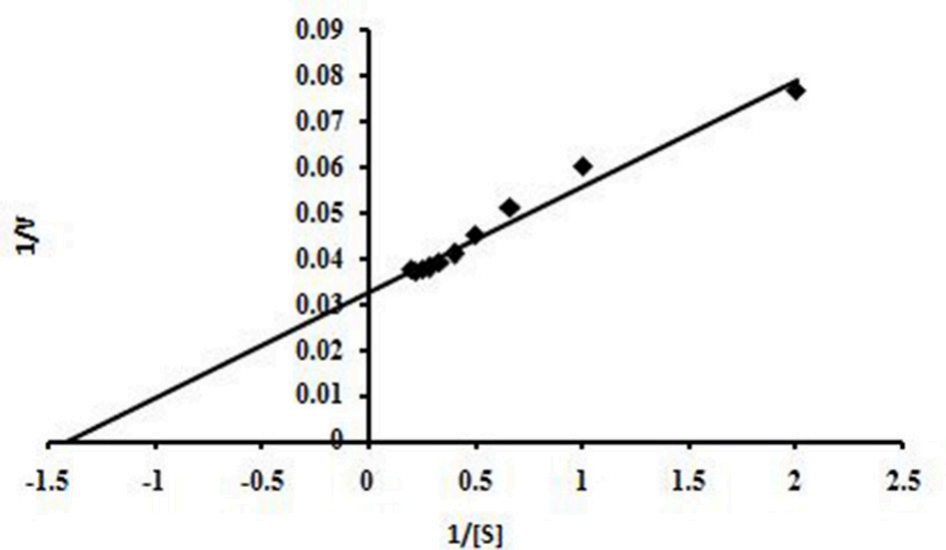
Graphic representation of Lineweaver-Burk determining the Vmax and Km values of xylanase *Anoxybacillus kamchatkensis* NASTPD13 when acted on Beech wood xylan.

This enzyme obeyed Michaelis–Menten kinetics with regard to beech wood xylan and based on a Lineweaver-Burk plot, the Km and Vmax values of NASTPD13 xylanase were 0.7 mg/ml, 66.64 μmol/min/mg respectively (Figure 10). Low Km value shows that xylanase has better affinity toward beech wood xylan.

### Shelf life of EnzymE

**Figure 11:**
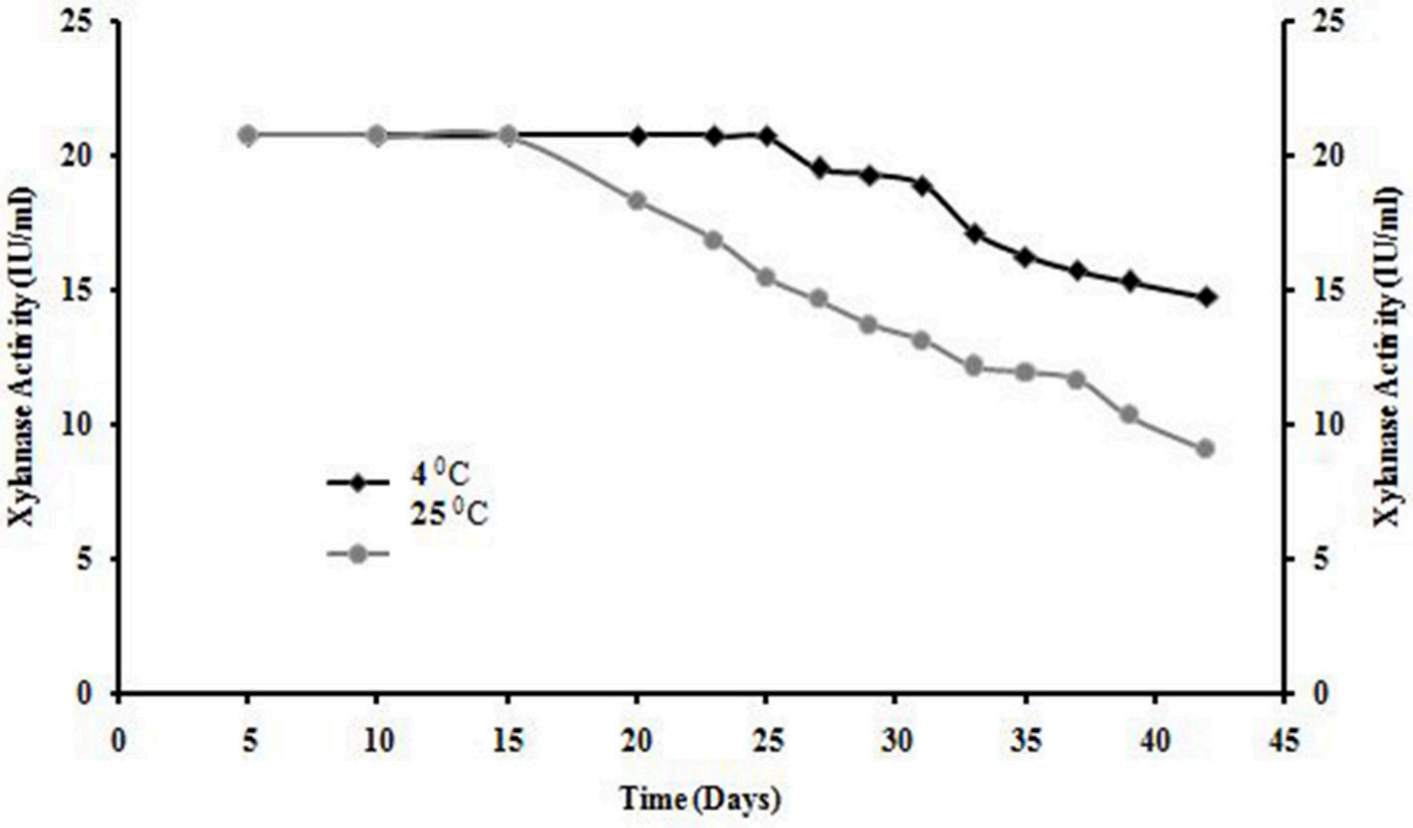
Shelf life of *Anoxybacillus kamchatkensis* NASTPD13 xylanase at 4°C and room temperature.

Shelf life of xylanase from *A. kamchatkensis* NASTPD13 was stable at 4°C for 25 days after that decline was observed but enzyme obtained 70% of its initial activity after 6 week. Whereas enzyme was completely stable at room temperature for 15 days but also showed 40% of its initial activity after 6 week (Figure 11).

## Discussion

This study dealt with production, purification and characterization of xylanase from thermophilic bacilli *A. kamchatkensis* NASTPD13 (KY373247). This is the first report on isolation and characterization of xylanase from *Anoxybacillus* strain from Paudwar hot springs, Nepal. The previous study by Yadav et al. (2017a) describes the biochemical, morphological and molecular characterization of *A. kamchatkensis* NASTPD13. Strain NASTPD13 was Gram-positive (b) with terminal spore-forming rod-shaped morphology (Figure 2B), able to grow at a temperature of 60°C. Therefore, they could be classified as thermophilic bacteria according to Brock (1978). Morphological and microscopic characteristics of NASTPD13 were similar to the characteristics of the genus Bacillus, as was described by Gordon et al. (1973) and Kristjansson (1991). For maximum xylanase production, optimization was done using various parameters. The optimized media composition and condition was (g/l) Beech wood xylan 10, yeast extract 5, peptone 5, K_2_HPO_4_ 1, MgSO_4_7H_2_O 0.2. The pH of the medium was 7.0 and the cultures were incubated at 60°C for 24 h in an orbital shaker (200 rpm). The study showed that after 24 h of incubation there was a decrease in xylanase production by *A. kamchatkensis* NASTPD13. *Anoxybacillus* sp. I3, B9.3, I4.1, I4.2, C26, ACT2 Sari, ACT14, BT2.1, and CT1Sari isolated from hot spring of Turkey all were reported to produce maximum xylanase at the end of 24 h (Inan et al., 2011). The growth of bacteria, as well as many enzymatic reactions are strongly influenced by pH via affecting enzyme structure and transport of ions, metabolites, and enzymes across the cell membrane (Liang et al., 2010). Optimum xylanase activity near neutral pH has been reported earlier for *Anoxybacillus pushchinoensis* A8 (Kacagan et al., 2008), *Anoxybacillus flavithermus* TWXYL3 (Ellis and Magnuson, 2012), and *Anoxybacillus rupiensis* (Derekova et al., 2007). Optimum growth temperature of 60°C for xylanase production has been also reported for *Anoxybacillus gonensis* (Belduz et al., 2003), *Anoxybacillus mongoliensis* (Namsaraev et al., 2010), and *Anoxybacillus* sp. WP06 (Peng et al., 2008). The higher growth temperatures (above 50°C) of the thermophilic bacteria reduces the risks of mesophilic microbial contamination (Yeoman et al., 2010). Above 1% xylanase showed decreased in xylanase production by NASTPD13 which might be due to the increase in the viscosity of the growth medium and preventing uniform nutrient and oxygen circulation, which attenuate the microbial growth thus decrease the xylanase production (Karim et al., 2015). *Anoxybacillus* sp. reported by Inan et al. (2011), and *Geobacillus* sp. strain WSUCF1 (Bhalla et al., 2015) also produced maximum thermostable xylanase when grown in production medium containing 1% xylan. Peptone and yeast extract are complex nitrogen sources with various growth factors and enhance the bacterial growth and enzyme production (Bibi et al., 2014). Further detail study on growth curve *vs* xylanase activity of NASTPD13 xylanase showed that the stationary phase started faster than reported previously for *Bacillus licheniformis* 7-2 (Damiano et al., 2003) and *B. licheniformis* SVD1 (van Dyk et al., 2009). Stationary phase of *Anoxybacillus* sp. 3M began at 96 h when grown in Brewers' spent grain (BSG) medium (Alves et al., 2016) and of *B. licheniformis* JK7 at 16 h when grown in Luria Bertani medium containing 1% CMC. The difference in growth phase seen between *A. kamchatkensis* NASTPD13 and other *Anoxybacillus* sp may be due to differences in culture conditions, amount of inoculums used and the differences in the species of *Anoxybacillus* (Seo et al., 2013). The decrease in xylanase activity might be due to the production of toxic metabolites during microbial growth which inhibits the enzyme synthesis (Irfan et al., 2016) or feedback inhibition caused by the high yield of end product-xylose produced from degradation of xylan (Bibi et al., 2014). There was increase in enzyme production with an increase in cell growth, which suggested that that xylan was actively utilized by *A. kamchatkensis* NASTPD13 during the growth phase. Similar time course of xylanase production was reported in *G. stearothermophilus* KIBGE-IB29 (Bibi et al., 2014). *A. kamchatkensis* NASTPD13 was able to produce xylanase in a short time period as compared to other strains of *Anoxybacillus* sp. strain I3, CT1Sari, BT2.1, I4.2, B9.3, I4.1, ACT2Sari, AC26, and ACT14 isolated from hot springs of Turkey (Inan et al., 2011)*, A. flavithermus* TWXYL3 (Ellis and Magnuson, 2012), *Anoxybacillus sp*. IP-C (Hauli et al., 2013), *Anoxybacillus* sp. 3M (Alves et al., 2016), *Bacillus pumilus*VLK-1 (Kumar et al., 2014), *B. subtilis* BS04, and *B. megaterium* (Irfan et al., 2016). Given *A. kamchatkensis* NAST-PD13 that takes short time to produce optimal xylanase enzyme levels, it should be considered as quite a favorable microbe for industrial production of xylanase.

Endoxylanases and β-xylosidases are the main enzymes for the hydrolysis of xylan fraction of biomass to monomeric xylose (Ye et al., 2017). NASTPD13 was able to degrade Beech wood xylan to xylooligosaccharide and xylose that suggest the presence of complete xylan degradation pathway in the strain. Various studies on *Anoxybacillus* have reported for production of either xylanase or β-xylosidase and complete degradation of xylan to monomer (xylose). The TLC image (Figure 6) showed that NASTPD13 xylanase was able to degrade xylan to xylo-oligosaccharide and monomeric pentose sugar xylose.

The molecular masses of various xylanases purified from different *Anoxybacillus* sp. ranged from 38 to 92 kDa (Table 2) and exhibited different biochemical characteristics (Goh et al., 2013). The 37 kDa of xylanase purified from the NASTPD13 culture medium during the exponential growth phase. The low-molecular-weight xylanase are useful in paper and pulp industries, because smaller enzymes penetrates the fiber wall structure easily which alters the pulp properties more efficiently. The hydrolytic pattern on zymogram suggests that this enzyme is involved in the hydrolysis of the xylan backbone because the Congo red dye interact only with (1,3- and 1,4-) ß–D-glucans (Kubata et al., 1995).

**Table 2:** Comparison between xylanases of different strains of *Anoxybacillus* species.

**S. N**.	**Microorganism**	**Molecular Weight (kDa)**	**Optimum Temperature (°C)**	**Optimum pH**	**References**
1	*A. kamchatkensis* NASTPD13	37	65	9	This study
2	*A. pushchinoensis* A8	83	55	6.5	Kacagan et al., 2008
3	*Anoxybacillus* sp. Ip C	45	70	9	Hauli et al., 2013
4	*Anoxybacillus* sp. E2	38.8	65	7.8	Wang et al., 2010
5	*A. flavithermus TWXYL3*	25–75	65	6	Ellis and Magnuson, 2012
6	*Anoxybacillus* sp. 3M	400-500	60	5.3	Alves et al., 2016

Enzymes display their maximum activity at their respective optimum conditions, deviations from the optimum cause a reduction of the activity (Bisswanger, 2014). At optimum pH the catalytic site is at ionization level. The chemical reaction is also strongly affected by temperature; because the fluctuation can affect the integrity of the secondary, tertiary, and quaternary structure of enzyme protein, which then will affect the enzymatic activity (Meryandini et al., 2006). NASTPD13 xylanase had an optimum condition at pH 9 and temperature of 65°C. Alkaline condition favored xylanase activity of *A. kamchatkensis* NASTPD13. Too high (over 9.0) or low (below 6.0) pH conditions significantly inhibited xylanase activity. This behavior is relatively similar to that described for xylanase from *Anoxybacillus* sp. strain I3, CT1Sari, BT2.1 ACT2Sari, AC26 (Inan et al., 2011) and *A. flavithermus* TWXYL3 (Ellis and Magnuson, 2012). *A. kamchatkensis* NASTPD13 xylanase retained 53.95% activity at its optimal pH 9 whereas comparing to the *A. flavithermus* TWXYL3 xylanase showed only 39% of activity at pH 9 (Ellis and Magnuson, 2012) and *A. pushchinoensis* A8 xylanase showed 91–96% activity at its optimum pH of 6.5 (Kacagan et al., 2008). When beechwood xylan was used as substrate, the Km and Vmax values of NASTPD13 xylanase were lower to that of *Saccharopolyspora pathumthaniensis* S582 (Km 3.92 mg/mL and Vmax 256 mmol/min/mg) (Kanokkorn Sinma et al., 1957), *Aspergillus ficuum* AF-98 (Km 3.267 mg/ml and Vmax 18.38 M/min/mg) (Lu et al., 2008), and *Bacillus amyloliquefaciens* (km 5.6 mg/ml and Vmax 433 μL/min/mg; Kumar et al., 2017). Whereas comparing within the Anoxybacillus sp., in presence of oat splet xylan *A. pushchinoensis* A8 showed (Km 0.909 mg/ml and Vmax 59.88 U/min/mg) (Kacagan et al., 2008) which were somewhat similar to NSTPD13 xylanase and *Anoxybacillus* Sp. Ip-C showed Km and Vmax value of 4.59 mg/ml and 13.5 μmol/min/mg respectively. These differences in the Km and Vmax values were may be due to different xylans substrate or assay conditions (Poorna, 2011) The value of Vmax is in favor of beech wood xylan, the small Km value in our study shows that the purified xylanases have high affinity for the substrate and this is of significance in industrial use of the enzyme. The shelf life of enzyme was also high enough which would be important for its application. Hence, it could be concluded that NASTPD13 xylanase could tolerate alkaline conditions and is possibly classified as an alkaline xylanase. Previous studies on the properties of *Anoxybacillus* xylanases and also data of this study showed that the xylanases of these bacteria are thermally stable. NASTPD13 xylanase possesses good stability at temperatures below 65°C and are rapidly inactivated at 70–80°C. In particular, thermostable and alkali-stable xylanases are more beneficial because it saves cooling cost and time (Demain et al., 2005; Liang et al., 2010). In this regard, the xylanase described herein works better at high pH and temperatures and it is best suited for industrial purposes. *A. kamchatkensis* NASTPD13 xylanase in comparison with xylanases of its neighbor species (Table 2) produced low molecular mass (37 kDa) xylanase and found more stable at both in higher temperature and alkaline pH. Therefore, *A. kamchatkensis* NASTPD13 xylanase can be a desirable enzyme for various biotechnological applications. The promising results can be exploited further for production of biotechnological important and industrially thermostable enzymes. This study also widens the opportunities for further research to be conducted to explore more the immense significance of these strains, especially for the industrially important enzymes.

## Conclusion

Thermophilic bacteria, *A. kamchatkensis* NASTPD13 has been characterized by various techniques including 16s RNA sequencing. *A. kamchatkensis* cultures at 60°C, pH 7 and in the presence of nitrogen sources produce a secretory xylanase enzyme. The secretory xylanase of *A. kamchatkensis* has been isolated, purified and characterized. The enzyme exhibits a molecular mass 37 kDa, has an optimum activity at pH 9.0 and 65°C. The enzyme is thermally stable and active in the alkaline range. As compared to the xylanase enzymes isolated from various *Anoxybacillus* species (Table 2), the xylanase of *A. kamchatkensis* NASTPD13 is unique, i.e., it has a lower molecular mass, thermostable and active in alkaline pH range. Above findings suggested that, *A. kamchatkensis* NASTPD13 xylanase can work under the harsh industrial conditions, i.e., high pH and temperature. Due to its potential applications in industrial processes, *A. kamchatkensis* NASTPD13 xylanase can be a novel industrial enzyme. Accordingly, it is a good candidate for various biotechnological applications including saccharification of hemicellulose and industrial pulping.

## Author contributions

JM, TB, LS, SK, GP, and GS involved in the study design. PY carried out the experiments and wrote the manuscript. All authors read and approved the final manuscript.

### Conflict of interest statement

The authors declare that the research was conducted in the absence of any commercial or financial relationships that could be construed as a potential conflict of interest.
